# Food-borne bacterial pathogens in marketed raw meat of Dharan, eastern Nepal

**DOI:** 10.1186/s13104-018-3722-x

**Published:** 2018-08-29

**Authors:** Kamana Bantawa, Kalyan Rai, Dhiren Subba Limbu, Hemanta Khanal

**Affiliations:** 1 Department of Microbiology, Central Campus of Technology, Tribhuvan University, Dharan, Nepal; 2 Department of Microbiology, Birendra Memorial College, Tribhuvan University, Dharan, Nepal

**Keywords:** Meat, Total viable count, Coliforms, Dharan

## Abstract

**Objectives:**

This study aims to assess the bacteriological quality of marketed raw meat with a special emphasis on isolation of *Escherichia coli*, *Salmonella* spp., *Shigella* spp., *Vibrio* spp., *Pseudomonas aeruginosa* and *Staphylococcus aureus* in raw meat marketed in Dharan. Altogether 50 meat samples were collected from local markets of Dharan and transported to the microbiology laboratory at 4 °C. The meat samples were homogenized in a sterile glass homogenizer and the possible pathogens were isolated and identified by conventional microbiological techniques.

**Results:**

The mean total viable count values were found having a mean count of 8.22 ± 0.14, 8.29 ± 0.17, 7.87 ± 0.18 and 7.92 ± 0.19 in terms of log_10_ CFU/g ± Standard Error for chicken, pork, buffalo, and goat meat respectively. Coliforms were found in 84% samples, *S. aureus* was found in 68% samples, *Salmonella* spp. in 34% samples, *Shigella* spp. in 6% samples, *Vibrio* spp. in only 3 samples and *P. aeruginosa* was isolated from 40% sample. Higher microbial load and presence of intestinal commensals *E. coli*, *Salmonella* spp., *Shigella* spp., *Vibrio* spp indicates that meat might be contaminated by the visceral content and consumers are at risk of getting a foodborne disease when eaten raw.

## Introduction

The term meat refers to the flesh, skeletal muscle and any attached connective tissue or fat excluding bone and bone marrow [1]. Meat is a good source of protein, essential fatty acids, minerals and vitamins but easily perishable because it provides the suitable medium for the growth of various microorganisms [1, 2]. The muscles of healthy animals contain less or nil microorganisms though, meat can be contaminated during slaughtering and transportation [3]. Contamination of raw meat easily occurs from external sources during bleeding, handling, and processing via knives, tools, clothes, hands, and air [4]. The contaminated meat and meat products readily cause a variety of biological, chemical, physical, and particularly microbial food hazards [5, 6]. The extent of microbial contamination and composition of microbial flora reflect the standard hygiene of meat [7].

Foodborne pathogens are the leading causes of illness and death in less developed countries [8]. Nearly 1.4 million cases are caused by nontyphoidal *Salmonella* serotypes and 270,000 cases are caused by pathogenic *Escherichia coli*, including *E. coli* O157: H7 [7, 8]. Although these pathogens usually cause self-limiting gastroenteritis, invasive diseases and complications also may occur. Similarly, systemic Salmonellosis infections can be life-threatening, and Shiga toxin-producing *E. coli*, particularly *E. coli* O157: H7, can cause bloody diarrhoea and hemolytic uremic syndrome [9].

Nowadays, the awareness has been growing on the public health impact of zoonotic foodborne pathogens transmitted from animal originated food [10]. The most important foodborne bacterial pathogens associated with meat are *Salmonella* spp., *Staphylococcus aureus*, *Escherichia coli*, *Campylobacter jejuni*, *Listeria monocytogenes*, *Clostridium perfringes*, *Yersinia enterocolitica* and *Aeromonas hydrophila* [11]. Among them, *Salmonella* species, *Campylobacter jejuni*, *Listeria monocytogenes* and verocytotoxin producing *E. coli* O157 are a major public health problem [9]. Additionally, *Pseudomonas* species are associated with spoilage of meat causing off-odours, off-flavours, discolouration and gas production [12, 13]. Similarly, *Vibrio* species are the leading cause of gastroenteritis, wound infection and septicemia in human [14, 15].

The marketed raw meat is available with objectionable hygiene and in the open air without adequate temperature control in Nepal. The present study was done to determine the prevalence of foodborne pathogens with special emphasis on *Salmonella* spp., *Staphylococcus aureus*, *Escherichia coli* and *Vibrio* spp. in marketed raw meat available in Dharan, eastern Nepal.

## Main text

### Methods

#### Study site

The study area is a city of Province No. 1 of Nepal and located in foothills of Mahabharat range in the north and Terai region in the south at the altitude of 349 meters. It is a trade centre between the hilly region and terai plains of eastern development region. The per capita meat consumption in Dharan is comparatively higher i.e. 13 kg whereas national per capita meat consumption is only 9 kg.

#### Analytical methods

A cross-sectional study was carried out in 6 months from January to June 2017 focusing on retail meat shops of Dharan city. A total of 50 (15 chicken, 15 pork, 10 buffalo and 10 goat meat) samples were collected. A sample of 250 g was collected in a sterile plastic bag and transported to the laboratory maintaining cold chain. The samples were collected at 7–9 a.m. Samples were processed immediately as soon as possible otherwise preserved at 4 °C.

Along with sample collection and homogenization, all of the analyses including total viable count (TVC), total coliform count (TCC), isolation of *E. coli*, *Salmonella* spp., *Shigella* spp., *Vibrio* spp., *Staphylococcus aureus* and *Pseudomonas* spp. were done as described by U.S. FDA guideline of bacteriological analytical methods for food samples [16]. The triplicate plate cultures were prepared for all tests performed and all of the culture media were used supplied from Himedia, India. Obtained isolates were identified by cultural characteristics, Gram staining, and biochemical tests as described by Bergey’s Manual of Determinative Bacteriology [17].

For statistical analysis, data obtained were tabulated with Microsoft Office Excel 2007 and analyzed with SPSS version 23. Chi square test was used to determine the significant association of dependent variables at 5% level of significance.

### Results and discussions

The mean value of TVC and TCC of raw meat is presented in Table 1. The mean TVC value was slightly higher in chicken (log._10_ 8.22 ± 0.14 CFU/g) and pork (log_10_ 8.29 ± 0.17 CFU/g) in compared to buffalo (log_10_ 7.87 ± 0.18 CFU/g) and goat (log_10_ 7.92 ± 0.19 CFU/g).

**Table 1:** Mean total counts of raw meat in log_10_ CFU per gram ± standard error (SE)

Sample (n = 50)	Mean TVC	Mean TCC
Chicken (n = 15)	8.22 ± 0.14	8.13 ± 0.13
Pork (n = 15)	8.29 ± 0.17	6.16 ± 0.92
Buffalo (n = 10)	7.87 ± 0.18	6.31 ± 1.06
Goat (n = 10)	7.92 ± 0.19	6.37 ± 1.07

Results revealed that the highest TVC was found in pork sample and the lowest TVC was found in buffalo meat sample. The microbiological condition of fresh raw meat of local market of Dharan can be assumed to be heavily contaminated with spoilage and pathogenic organisms. In the case of coliforms, the lowest TCC was found in pork meat (6.16 ± 0.92 CFU/g) and the highest coliform count was found in chicken meat (8.13 ± 0.13 CFU/g). From statistical analysis, a significant association between the type of meat and the coliform count was not observed (P > 0.05).

Total viable count was found to be higher in almost all of the samples than the inspected German Quality meat standards, microbiological standards of Europe and United States, EU microbiological standard of cut meat and Oregon state microbiological standard for fresh meat. The result obtained was also higher than guideline by ISO, being followed by Nepal, which has set 105–107 total viable count and absence of coliforms in 0.01 g of raw meat and ≤ 104 total viable counts and absence of coliforms in 0.01 g for pre-cooked meat. The reason behind the higher prevalence rates could be could be related to the difference in time and season of research.

Table 2 illustrated that the prevalences of different bacterial pathogens. The highest prevalence of *Salmonella* spp., *E. coli,* and *P. aeruginosa* were found in chicken meat. Neither of chicken meat was contaminated with *Vibrio* spp. and neither of buffalo meat was contaminated with *Shigella* spp. From the statistical analysis, it can be concluded that there was no significant relationship between the type of meat and the presence of *S. aureus* (P > 0.05), *E. coli* (P > 0.05), *Shigella* (P > 0.05), *Vibrio* (P > 0.05) and *P. aeruginosa* (P > 0.05). However, there was a significant association between the type of meat and *Salmonella* spp. (P < 0.05).

**Table 2:** Prevalence of pathogens in raw meat

Type of meat	Prevalence of pathogens (%)					
	*S. aureus*	*E. coli*	*Salmonella* spp.	*Shigella* spp.	*Vibrio* spp.	*P. aeruginosa*
Chicken (n = 15)	53.33	66.6	60	4	0	46.66
Pork (n = 15)	73.33	60	10	0	2	40
Buffalo (n = 10)	80	40	20	2	2	40
Goat (n = 10)	70	46.7	33.3	0	2	33.33
Total (N = 50)	68	54	34	6	6	40
P-value	0.501	0.527	0.047	0.346	0.674	0.708

In this study, *S. aureus* was found in 68% samples. This prevalence is higher than previous researches by Tassew et al. in Ethiopia in 2010 and Abdalrahman et al. in beef [18, 19]. The prevalence of *S. aureus* was also greater than the study of Rong et al. in retail foods of China [20]. The higher prevalence of *S. aureus* indicates that inadequate cleaning, unsatisfactory handling, and post-processing contamination from the polluted atmosphere around shops. High prevalence of *S. aureus* in raw meat and handlers contain health hazards like toxin-mediated virulence and invasiveness to consumers [21]. These higher prevalence rates could be due to hand evisceration technique and inadequate hand washing.

As an indicator of hygiene and sanitary quality, the presence of *E. coli* suggests that consumers are at risk of being food poisoned and the presence of other pathogenic flora [22]. *E. coli* was found in 54% of the total samples. The prevalence of *E. coli* was also higher than in previous studies of Roades et al. in 2008, Lee et al. in 2006–2012 and Rontsiou et al. in 2012 [23–25].

Another potential pathogen *Salmonella* spp. was found in 34% of total samples which is higher than previous studies by Tassew et al. in 2010 and Garedew et al. in 2015 [14, 18]. The prevalence of *Salmonella* spp. in meat was beyond the EU Microbiological standards of cut meat for sale and further processing according to which *Salmonella*. Differences in prevalence rates from this study to another might be attributed to the unhygienic processing and poor sanitation of meat shops. It showed that meat retailers were found to be unaware of the basic requirements of basic guidelines regarding meat. It had been proved that direct contact with raw meat might pose health hazards to humans especially butchers because *E. coli*, *Salmonella*, *Shigella* and *Vibrio* are transmitted via the faecal–oral route.

*Shigella* spp. were found in 6% samples which is higher than the research by Tassew et al. in which it was found in only 0.6% samples but lower than Garedew et al. in 2016 in Ethiopia [14, 26]. Similarly, *Vibrio* spp. were also found in 6% samples which is lower than the research by Lopatek et al. in 2015 and Yucel et al. in 2010 in marine seafood [27, 28]. *Pseudomonas aeruginosa* was isolated from 40% sample which was comparable to findings of researches by Kwan et al., Gennari et al., Arnautet al., and Elmanhdi et al. at different dates and locations [29–32]. Higher prevalence of *Pseudomonas aeruginosa* indicates the post-processing contamination and meat are prone to spoilage. The presence of different pathogens in examined samples represents a great public health risk. Our results concluded that the cross-contamination occurs from the raw meat to handlers to consumers.

#### Conclusions

All the meat samples were found to contain higher microbial load than prescribed standards. This study showed that 68% of samples were contaminated with *S. aureus*, 54% samples with *E. coli*, 34% samples with *Salmonella* spp., 40% of samples with *Pseudomonas* spp. and 6% samples with *Vibrio* spp. and *Shigella* spp. Presence of intestinal commensals *E. coli*, *Salmonella* spp., *Shigella* spp., *Vibrio* spp. indicates the alarming public health concerns, which needs to be studied in larger contexts to identify the source of contamination. This study also revealed that unhygienic processing and poor sanitation of meat shops and meat retailers were unaware of basic requirements and guidelines of meat shop. To upgrade the quality of raw meat, routine monitoring of meat shops, implementation of Animal Slaughter and Meat Inspection Act 2055 and awareness campaign for the butchers and consumers should be done.

## Limitations

Confirmation of bacteria by molecular methods was not performed. Similarly, serotyping of isolated pathogens were not done. Antibiotics susceptibility patterns of isolates were not examined.

## Abbreviations

CFU: colony forming unit
FDA: Food and Drug Administration
SE: standard error
TVC: total viable count
TCC: total coliform count
